# Chromatin Proteins: Key Responders to Stress

**DOI:** 10.1371/journal.pbio.1001371

**Published:** 2012-07-31

**Authors:** Karen T. Smith, Jerry L. Workman

**Affiliations:** Stowers Institute for Medical Research, Kansas City, Missouri, United States of America

## Abstract

Environments can be ever-changing and stresses are commonplace. In order for organisms to survive, they need to be able to respond to change and adapt to new conditions. Fortunately, many organisms have systems in place that enable dynamic adaptation to immediate stresses and changes within the environment. Much of this cellular response is coordinated by modulating the structure and accessibility of the genome. In eukaryotic cells, the genome is packaged and rolled up by histone proteins to create a series of DNA/histone core structures known as nucleosomes; these are further condensed into chromatin. The degree and nature of the condensation can in turn determine which genes are transcribed. Histones can be modified chemically by a large number of proteins that are thereby responsible for dynamic changes in gene expression. In this Primer we discuss findings from a study published in this issue of *PLoS Biology* by Weiner et al. that highlight how chromatin structure and chromatin binding proteins alter transcription in response to environmental changes and stresses. Their study reveals the importance of chromatin in mediating the speed and amplitude of stress responses in cells and suggests that chromatin is a critically important component of the cellular response to stress.

## Organisms Respond to Stress in Multiple Ways

Stresses in the environment may include temperature changes, chemical toxins, nutrient deprivation, pathogens, or even threats by predators; the immediate response to these stresses is critical for survival. However, how these intricate responses are orchestrated remains a mystery. In multicellular organisms, the response is inherently complex and involves communication between multiple cell types and tissues. Initial studies have accordingly focused on the molecular responses to stress in single celled organisms, such as yeast. Each yeast cell is particularly susceptible to environmental changes and is primed to respond to a wide variety of changes, such as altered nutrient availability or the presence of toxins [1],[2].

Many molecular events collaborate in the response to environmental changes, and the underlying mechanisms are conserved from yeast to humans. The first layer of the response involves a sensor, which is necessary to relay the message that there is a stressor present and that the cells need to adapt [2],[3]. Sensors may be extracellular to respond to external stimuli or intracellular to respond to alterations in cellular homeostasis. Some examples include heat shock factors, oxidative stress responsive factors, and specific nutrient sensing factors [2]–[7]. Interestingly, many of these factors turn out to be involved in transcription regulation.

One of the best studied examples is the heat shock response that is initiated largely by heat shock factor 1 (HSF1) [5],[6]. The heat shock response is often initiated following an increase in temperature, but can also respond to other stressors of cellular viability and is found in bacteria, plants, yeast, as well as humans. Unusual heat increase in cells can lead to the misfolding of proteins, which can affect the normal activity of proteins and result in disease [8]. Through a still poorly understood mechanism, HSF1 is induced to translocate to the nucleus from the cytoplasm in response to protein misfolding. HSF1 then turns on a transcriptional program that induces multiple additional heat shock proteins (protein chaperones) to fix or replace the misfolded proteins [5],[6].

In addition to HSF1, there are other surveillance mechanisms in the cell that maintain proper protein folding. The detection of unfolded proteins also occurs in the endoplasmic reticulum (ER), which is responsible for assembling and folding many proteins in the cell [7]. One of the main sensors of the unfolded protein response (UPR) is a protein called IRE1, which can directly detect and bind to unfolded proteins [7],[9],[10]. IRE1 becomes activated once it binds to unfolded proteins, and this activation turns IRE1 into an effector protein that relays the stress messages in the cell [7],[9],[10]. Thus, this process as a whole also requires many additional intracellular responses, including alterations in transcription.

**Figure 1:**
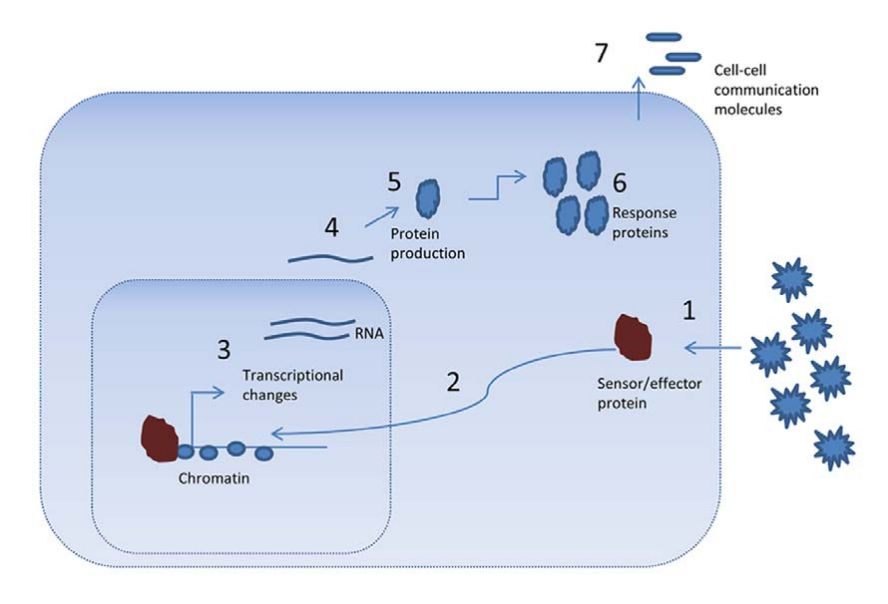
Cellular responses to stress and environmental factors. Stresses such as heat shock are sensed by factors located inside of or outside of the cell (1). In the case of HSF1, it relays the message to the nucleus (2) to strongly increase the transcription of genes involved in fixing protein shape (3). RNA stability (4) and protein production levels (5) are also important factors determining the response to stress. Protein activity (6), such as the chaperones induced by heat shock, is critical in mediating the response. In higher eukaryotes, cells may send signals (7) to neighboring cells to assist in mounting a larger stress response encompassing many cells and tissues.

The stress responses inside cells are complex processes, which can be critical for survival and are tightly controlled at several levels, including transcription. Figure 1 summarizes some of the steps inside cells that can help cells and organisms respond to stress, including the heat shock response and the unfolded protein response. These include affecting the transcription and translation of a variety of genes, as well as altering the stability of the RNAs and proteins [5]. Other important factors are the activity of proteins, such as the ability of HSF1 to translocate to the nucleus, the ability of the sensor to recognize the stressor, and crosstalk among proteins (Figure 1). Responses to stress can feed into the cell and trigger signaling cascades, many of which can feed back to steps anywhere in the pathway [3],[5],[11]. Although the orchestration of these steps is complex, most healthy cells appear to be able to coordinate them without fault.

One underlying theme in many responses to stress is that at least in part, they are controlled by altering the transcription of genes. Recent articles have addressed other levels of regulation and we refer the readers to some examples for further explanation [5],[12]–[14]. The expression of genes in response to environmental changes has been studied in various facets of biology. For example, alterations in gene expression are seen upon viral or bacterial infection to assist in mounting immune responses [15]–[17]. Exposure to DNA damaging agents, such as UV radiation, can also affect gene expression and the activity of transcriptional regulators [18],[19]. Even worms, when environmental conditions are harsh, turn on a transcriptional program that allows them to “freeze time” until conditions are favorable for reproduction [20]. Though all of these changes require many steps, there are usually critical transcriptional regulators that play roles in these processes.

## Chromatin Allows Flexibility in the Packaging and Expression of Genes

**Figure 2:**
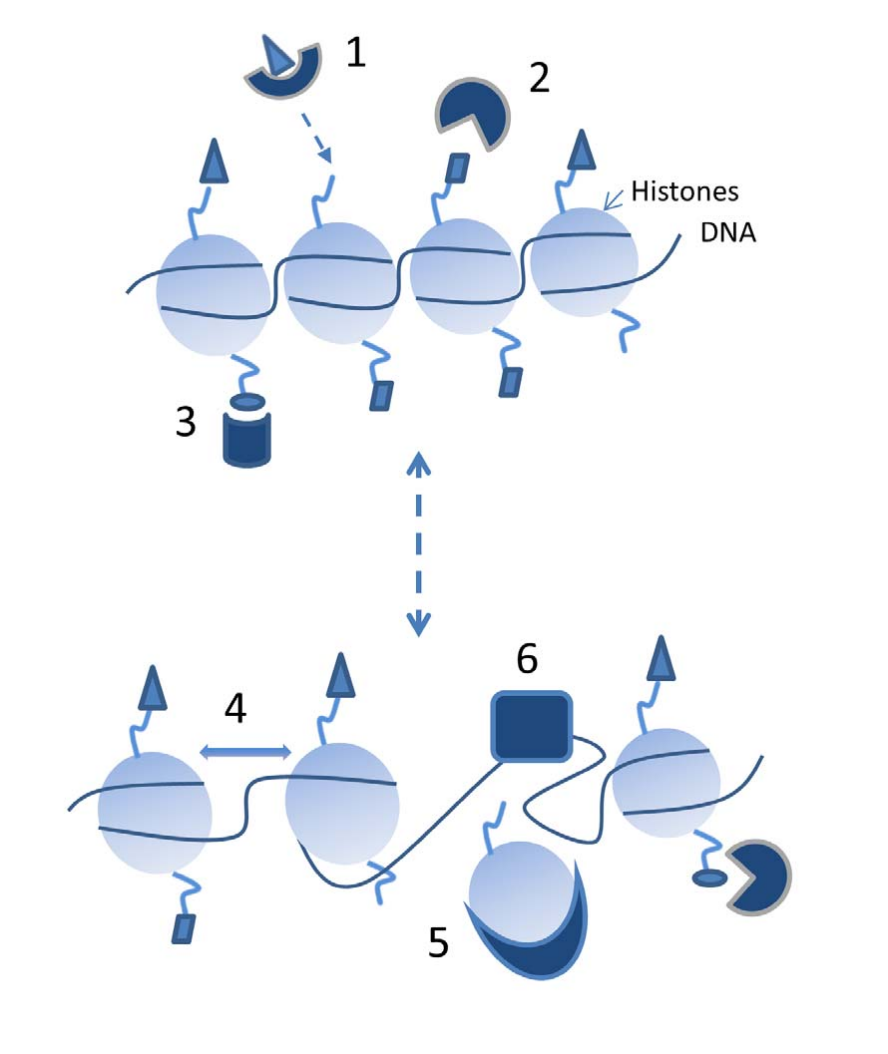
Chromatin structure and binding proteins can affect transcription through multiple avenues. Chemical additions can be made to histones by chromatin-modifying enzymes such as Set1, which adds methyl groups to a specific place on the histone (1); many of these chemical modifications can be removed by other proteins (2). Some proteins bind to specific modifications on histones that have been added (3). Nucleosome spacing (4) can be altered by chromatin remodelers that use the power of ATP to drive movement and histone chaperones (5) can remove and replace histones on DNA. All of these factors can control access of the DNA and chromatin to other factors, such as transcription factors that may need to bind open pieces of DNA (6). This is a dynamic process requiring many proteins to act in concert as appropriate.

As mentioned above, in addition to transcription factors, histones and chromatin proteins are critical responders to stress that alter gene expression (Figure 2). Nucleosomes comprise four major histone proteins—Histone H3, H4, H2A, and H2B [21]—and are further packaged into higher order chromatin, often with the help of many non-histone proteins. This packaging not only serves to physically fit the DNA into the limited volume of the nucleus, but also acts as a principal regulator of transcription by acting in concert with transcription factors [22],[23]. Many chromatin-modifying proteins put specific chemical marks on histones and these marks can in turn be recognized by specific chromatin binding proteins that alter other aspects of chromatin structure or behavior [24]. Histone chaperones, for example, can add or remove histones from DNA, and chromatin remodeling enzymes can alter the degree of chromatin compaction [21],[25].

To identify a gene's function, scientists often mutate the gene in question and assess the outcome in the organism. These experiments are often carried out under a pre-defined laboratory condition, which is unlikely to exactly mimic that of the organism in its ever-changing natural environment. Thus perhaps it is not surprising that previous research revealed that despite the importance of chromatin for gene expression, some chromatin factors have minimal effects on transcription in steadily growing cells [26]. Thus it has been suggested that the functions of some chromatin enzymes may only be important during stress or “real-life” conditions [26]. Indeed there are several examples implicating chromatin in stress responses. Heat shock and oxidative stresses can alter how tightly the histones are held onto the DNA [27]–[29]. Also a number of stresses can change the localization or activity of chromatin modifying enzymes that in turn can cause alterations in the distribution of histone modifications across the genome [30]–[33]. From these and other examples, it is clear that at least some chromatin proteins are important in mediating stress responses.

## Stress Reveals New Insights into Chromatin Function

A study in this issue of *PLoS Biology* by Weiner et al. 34 tests the impact of hundreds of chromatin mutations on the transcriptional response to stress in yeast. As a stressor, the authors employ diamide, a compound known to induce cellular damage through the creation of reactive oxygen species (ROS). Using existing knowledge of diamide transcriptional effects on wild-type yeast [1], the authors design experiments to determine how the transcriptional stress responses to diamide compare across a variety of yeast mutants. The authors survey over 100 yeast strains with knock-out mutations for genes encoding proteins with known roles in chromatin biology. In addition, they analyze 83 yeast strains where histone residues are mutated, either to deliberately mimic a specific chemical modification or to prevent modification by the appropriate enzyme in the cell. Importantly, the authors also study these changes over time to gain valuable information regarding the kinetics of these stress-induced transcriptional outputs. In general, this study reveals many changes in RNA levels in histone and chromatin effector protein mutants that were not apparent while growing in unstressed conditions.

These findings suggest that chromatin plays an even more important role during stress response than it does under non-stressed conditions. The thoughtful experimental design of this study allows the authors to compare data from single mutants with each other and ask three questions: (1) Do distinct chromatin effector proteins share functional gene outputs in response to stress? (2) Do different histone marks have common roles in the stress response to diamide? and (3) Do the functions of some histone marks mimic those of specific chromatin modifying proteins? By coordinating the answers obtained from these comparisons, new insights into chromatin function during stress were revealed.

One of the major themes revealed is that specific chromatin modifications and proteins do not necessarily assume the same function under steady state conditions as they do under stress. For example, one protein (Set1) adds methyl groups to histone H3 at lysine 4 and was known from previous studies to have minimal effects on transcription during non-stressed conditions [26]. However, in response to stress, deletions of *set1* reveal a strong role for this protein in ribosomal gene repression. As expected, the effect on ribosomal gene repression also occurs when the histone residue that Set1 methylates (H3K4) is mutated. They couple this information with time-course studies of H3K4 methylation in stressed wild-type cells and learn that this mark increases in abundance at some repressed subsets of genes involved in ribosomal protein production and RNA maturation. This was unexpected since H3K4 methylation is often found in actively transcribed genes and hence was considered an “activating mark” [35]. The current study also reveals findings about other modifications and shows that H3K36 methylation, which was thought to be involved in regulating downstream areas of genes, can also be found at promoters under stress conditions [36]. Thus, some histone marks and proteins are particularly important during stress and they may take on alternative roles depending on the cellular environment.

Another finding is that many chromatin-related proteins affect the kinetics and amplitude of the gene expression response. Specifically, the authors found that proteins and histone modifications that affect nucleosome stability through modulation of histone turnover often affect how fast the genes respond or whether the overall transcriptional output is greater or smaller in magnitude. This suggests a very important role for chromatin structure in modulating the speed and overall intensity of the transcriptional response to stress. Lastly, there were many interesting observations regarding potential new pathways of chromatin and gene regulation as well as new proteins not previously thought to collaborate. Overall this study provides a comprehensive overview of chromatin function during the stress response, but also leads to several questions that we introduce below.

## Further Considerations

The ability of organisms to respond to environmental changes is critical for survival. Altering the activity of genes inside cells plays a large role in mediating these responses. The study by Weiner et al. 34 in this issue of *PLoS Biology* reveals that chromatin plasticity must be tightly regulated on a global level in yeast to mediate the transcriptional response to stress. This study reveals novel roles for specific chromatin proteins and new chromatin-related pathways. Importantly, it also suggests that chromatin “marks” may either be activating or repressing depending on the cellular context and environment, and that examination of chromatin under static conditions may have led to limited views on these roles.

Interestingly, their experiments reveal that after consistent exposure to diamide, the cells reach what appears to be a new steady-state, suggesting that cells may be adapting to the stress. This finding is consistent with previous studies and is especially interesting in light of work regarding the potential heredity of chromatin marks and transcriptional memory [1],[37]. Follow-up studies could address the status of the chromatin after stress removal to test if the cells return to their original steady-state levels of operation or if they remain at the new steady-state and for how long. Also, it will be of interest to see whether the genes respond faster in subsequent challenges with stress, such as has been observed for the *gal* genes in yeast [37].

Lastly, these chromatin-based experiments are particularly interesting regarding the importance of chromatin in multicellular organisms for the maintenance of healthy cells. Future steps may include testing the importance of specific mammalian chromatin proteins in handling diverse stress responses. Findings in this yeast study show that certain mutations heighten or dampen the responses to stress, suggesting that chromatin proteins could affect responses to certain drugs, either by making organisms more resistant or more sensitive. Since some chromatin proteins are known to be mutated in cancers and other diseases, it will be particularly important to address whether specific mutations affect the speed and robustness of therapeutic drug responses [38]. Such studies could have an impact on multiple aspects of disease biology.
